# Job stress and interpersonal relationships cross country evidence from the EU15: a correlation analysis

**DOI:** 10.1186/s12889-020-09253-9

**Published:** 2020-07-20

**Authors:** Nunzia Nappo

**Affiliations:** Department of Political Science, University of Napoli “Federico II”, Via Rodinò 22, 80134 Napoli, Italy

**Keywords:** Job stress, Interpersonal relationships, Europe, Ordered probit model

## Abstract

**Background:**

The aim of the study is to analyse the association between job stress and interpersonal relationships on and outside of the job in Europe. The main assumption of the paper is that since social relations at various levels enhance individual well-being, they may counteract stress created by an unfavourable work environment.

**Methods:**

The econometric analysis, based on a standard ordered probit model, employs data taken from the Sixth European Working Conditions Survey carried out in 2015 and released in 2017.

**Results:**

The results show significant correlations between interpersonal contacts on and outside of the job and job stress. Help and support provided by one’s manager decreases the probability of being stressed at work, while receiving help and support from co-workers is likely to increase the probability of job stress occurrence. However, maintaining cooperation and getting on well with colleagues decrease the probability of experiencing stress, confirming the positive and gratifying features of contact with co-workers reported by the literature.

**Conclusions:**

While we were not able to establish the direction of causality between job stress and interpersonal relationships (a limitation of this paper), the present work contributes new evidence to the literature on occupational stress. Our results show that interpersonal relationships on and outside of the job can be considered valuable resources that, when available to an individual, are useful for managing stress created by workplace stressors.

## Background

Job stress has been becoming a worldwide issue with consequences for employees, organizations, and economies [1]. The continuous and rapid transformation of work has rendered the demands of working life increasingly difficult: imbalances between perceived demands and employees’ resources and abilities can undermine workers’ wellbeing [2], causing stress. “Work-related stress is the response people may have when presented with work demands and pressures that are not matched to their knowledge and abilities and which challenge their ability to cope” [3]. Currently, job stress is among the problems with which employees are confronted more often [4] as a broad negative outcome of work life; as such, job stress has become a major social phenomenon and an issue of public health [5]. Concerned with the growth of job stress, academics in the fields of social sciences have debated its determinants and consequences. Sickness and subsequent absenteeism are some of the individual consequences of working in stressful work environments. Workers’ job stress diseases and absenteeism in turn affect the productivity and performance of organizations, which in turn affects national economies and society at large with substantial financial costs. For 2014, the cost of job stress within the EU-15 was assessed at €26.47 billion [6]. Such numbers reveal the existence of this phenomenon and the need for action.

The literature [3, 7, 8] has proposed several drivers of job stress, which include the physical environment, workloads, career advancement, management styles, working relationships, organizational support, work itself, rewards, job security, job autonomy, role conflict and ambiguity. “Stress occurs in a wide range of work circumstances but is often made worse when employees feel they have little support from supervisors and colleagues, as well as little control over work processes” [3]. In particular, social or physical segregation, poor relationships with superiors, interpersonal conflicts, and a lack of social support can be considered among the stressful characteristics of work [9].

The main aim of this paper is to study the link between job stress and interpersonal relationships on and outside of the job in Europe. Interpersonal contacts at various levels are likely to improve individual well-being [10], and for this reason, they could counteract stress coming from unfavourable work environments: social relations on and outside of the job are likely to moderate the negative effects of other psychosocial risks, the impacts of which are more marked when relationships provide little support [9, 11].

The empirical analysis employs data taken from the Sixth European Working Conditions Survey (EWCS6) released in 2017 [12]. The survey presents a varied picture of Europe at work over time and across countries, occupations, genders and age groups. The paper focuses on EU15 countries. The original contributions of the paper to the literature are twofold. First, we use EWCS6 data to analyse the association between job stress and interpersonal relationships. To the best of our knowledge, this is the first study to apply these data for such an investigation. Second, the paper considers a large group of countries (EU15) and, therefore, provides a broad account of the impact of social interactions on job stress across Europe.

The paper is organized as follows. Section 2 focuses on drivers of job stress. Section 3 describes the data and methodology used. Section 4 illustrates and discusses the results of the econometric analysis. The last section concludes.

### Drivers of job stress

Job stress is a psychosocial risk with adverse health outcomes (see, among others [13]). According to Cox and Griffiths [14], psychosocial risks can damage a worker’s psychological or physical well-being through interactions between the design and management of work. Psychosocial risks seem to be more difficult to manage than traditional physical risks [15] and may be important in causing illness [16]. Among health-related implications of job stress have been detected cardiovascular diseases, diabetes, musculoskeletal problems, depression or bipolar disorders [5–13, 17].

Job stress arises “when the demands of the work environment exceed the workers’ ability to cope with (or control) them” [18]. As stress has been analysed and continuously debated among experts, it is not easy to provide a definition for it. However, the idea that stress originates from a perceived imbalance in interactions between an individual, the environment and other individuals is generally accepted [19]. Stress is likely to arise when workers must deal with demands from co-workers/managers or from the physical or psychosocial environment to which they feel incapable to commensurably respond. Under such circumstances, the organism reacts to manage the situation. The nature of this reaction is affected by several factors, which can include the magnitude of demands made, personal features and coping strategies, limits placed on workers’ abilities to make decisions about their work, and support provided from others. In addition, specific work environments, tasks, organizations and cultural factors characterize drivers of work-related stress in different ways [5].

One of the theoretical frameworks within which it is possible to study job stress is the “job strain model”, which is also known as “the demand-control model” [20–22]. According to Karasek et al. [23], works associated with high psychological demands (regarding the pace, quantity and difficulty of work) and less control (limited abilities to schedule one’s own duties and to manage one’s skills) are related to high degrees of job strain and, in turn, with high levels of job stress. Stressful job circumstances as identified by the model involve high demands and low levels of control associated with low degrees of social support. More specifically, according to the “job-strain model” [20], it is possible to identify four types of jobs: 1) “high-strain jobs” with high psychological demands and low decision latitude; 2) “active jobs” for which even if the demands are high the workers have adequate control over their activities and they can also use available skills; 3) “low-strain jobs” with low psychological demands and high levels of control; 4) “passive job” with low demands and low control.

Johnson and Hall [24] and Karasek and Theorell [20] added “social support” to the “demand-control model” [21], turning the model into the “demand-control-support” model. According to the “demand-control-support” model, support provided by supervisors and co-workers is likely to buffer negative effects of high demands and low levels of control. Positive relationships with colleagues and superiors may alleviate (buffer) stressful situations [25, 26]. Cummins [27] stated that workers who maintain good relationships with their supervisors and co-workers are generally successful and productive at work, even when levels of job stress are high. Several studies (see, among others [17, 28]) show that social support is likely to become a positive resource for workers and find that support from co-workers and managers is important in moderating the effect of high emotional demands. This means that workers subjected to high emotional demands are likely more able to manage them when they have proper support from their colleagues. Given their role, supervisors can interact with employees and listen to their complaints, in turn helping their employees find resources to counteract their stress [29]. On the other have, co-workers can help colleagues complete work tasks, decreasing stress levels [30]. Other works [31, 32] show that interpersonal relationships (and personality factors) can weaken the effects of role ambiguity, role conflict, overload (quantitative and qualitative), withdrawal, low self-confidence, low job satisfaction and job tension on stress levels.

However, another strand of the literature [33, 34] states that the effects of interpersonal interactions on the job can sometimes be negative, rendering relationships with colleagues and supervisors a source of stress. In particular, interpersonal work relations may cause stress when conflicts arise among co-workers. Discord may arise due to numerous reasons, for instance, disagreements on how tasks should be performed. Conflicts can also be of an organizational (institutional policies) or intra-individual nature (this type of conflict can emerge when workers’ values clash with their job demands) and can involve role conflicts as well [34]. Stress can also arise from interpersonal relationships on the job, which occurs when employees experience team pressure and hold opinions not shared by their colleagues [35].

## Methods

Data drawn from the Sixth European Working Conditions Survey, carried out in 2015 and released in 2017, have been used for the econometric analysis. Data were accessed and downloaded via the UK Data Service. The survey offers an extensive account of Europe at work over time across countries, occupations, genders and age groups. The European Working Conditions Survey provides an outline of working conditions in Europe. A random sample of workers, roughly 43,000 employees aged 15 and over, have been interviewed face-to-face. The survey contains issues related to employment status, working time duration and organization, work organization, learning and training, physical and psychosocial risk factors, health and safety, work-life balance, worker participation, earnings and financial security, and work and health. The sample includes 35 countries, including the EU28, Norway, Switzerland, Albania, the Former Yugoslav Republic of Macedonia, Montenegro, Serbia and Turkey. There is not a panel dimension of the Survey (for a similar description of the Survey see also [36]).

The econometric analysis focuses on the following EU15 countries: Austria, Belgium, Denmark, Finland, France, Germany, Greece, Ireland, Italy, Luxembourg, the Netherlands, Portugal, Spain, Sweden, and the United Kingdom.

While the study focuses on employed workers, the survey records information on both employed and self-employed workers.

After removing unselected respondents and missing dependent and independent variables, the final dataset is a cross-sectional sample of 10,882 observations.

### Dependent variable

Although the “the demand-control-support model” implies other different indicators for the identification of the dependent variable, given the availability of information in the EWCS6 (2017), we selected as dependent variable “*job stress*”, which is a self-rated/subjective indicator of perceived stress and has been measured through individual interviews (i.e. the subjective perspective of the participants was asked for). Several doubts have been raised on the reliance of subjective measures of job stress (see for instance [37]). Measures of objective job stress are not easy or possible to obtain, while self-reported measures tend to be easily collected. However, both measures are likely to provide useful information [37, 38].

Job stress is assessed with the following item (Question n. 61 m of the EWCS6 Questionnaire): “You experience stress in your work. Please select the response which best describes your work situation”. Responses are expressed on a on a 5-point scale: 1 Always; 2 Most of the Time; 3 Sometimes; 4 Rarely; 5 Never. Table 1 shows descriptive statistics for the dependent variable.

**Table 1:** Job Stress Descriptive Statistics

	Total Percent	Male Percent	Female Percent
*Always*	10.31	10.25	10.37
*Most of the time*	16.88	16.49	17.27
*Sometimes*	39.32	37.90	40.67
*Rarely*	18.74	20.21	17.31
*Never*	14.75	15.15	14.38

### Independent variables

The selection of proper explanatory variables was driven by theory (the “demand-control-support” model) and particularly by our aim to study the association between job stress and interpersonal relationships on and outside of the job (most of the explanatory variables are the same employed in [36]). A number of standard socioeconomic control variables are included in the econometric analysis as well. Tables 2 and 3, respectively, provide a description of the independent variables used in the empirical model and descriptive statistics for the sample (for brevity, the tables do not include the 15 country dummies considered in the econometric analysis).

**Table 2:** Independent Variables: a definition

***Variable***	***Description***
*Demographic*	
Male	1 if male; 0 otherwise
Age1	Age in years at the time of the survey interview - 15/34 years, 0 otherwise
Age2	Age in years at the time of the survey interview - 35/54 years, 0 otherwise
Age3	Age in years at the time of the survey interview - 55/74 years, 0 otherwise
Age4	Age in years at the time of the survey interview - 75/89 years, 0 otherwise (reference group)
Has a spouse or a partner	1 if she/he has a spouse or a partner, 0 otherwise
Has a child	1 if she/he has at least one child and 0 otherwise
Low level of education	1 if highest level of education is primary education, 0 otherwise (reference group)
Middle level of education	1 if highest level of education is secondary education, 0 otherwise
High level of education	1 if highest level of education is tertiary education, 0 otherwise
Ends meet	How the interviewee household total monthly income is able to make ends meet (from 1 very easily to 6 with great difficulty)
*Job characteristics*	
Permanent job	1 if the employment contract has an unlimited duration, 0 otherwise
Part time job	1 if she/he works part time, 0 otherwise
How many hours1	N. of hours the interviewee usually works per week - 1/20 h, 0 otherwise (reference group)
How many hours2	N. of hours the interviewee usually works per week - 21/40 h, 0 otherwise
How many hours3	N. of hours the interviewee usually works per week - 41/50 h, 0 otherwise
How many hours4	N. of hours the interviewee usually works per week - 51/105 h, 0 otherwise
Health risk	1 if the worker thinks that her/his health or safety is at risk because of her/his work, 0 otherwise
Work affects health1	1 if her/his work affects health mainly positively, 0 otherwise
Work affects health2	1 if her/his work affects health mainly negatively, 0 otherwise
Work affects health3	1 if her/his work does not affect health, 0 otherwise (reference group)
External Contacts	1 if her/his work involves visiting customers, patients, clients or working at their premises or in their home, 0 otherwise
Work and Family	1 if her/his working hours fits in with her/his family or social commitments outside work, 0 otherwise
Trade Union	1 if within her/his company or organisation there is trade union, 0 otherwise
Job satisfaction	1 if she/he is satisfied with working conditions, 0 otherwise
*Relationships on the job*	
Colleagues Support1	1 if her/his colleagues help and support her/him (always, most of the time), 0 otherwise
Colleagues Support2	1 if her/his colleagues help and support her/him (sometimes), 0 otherwise
Colleagues Support3	1 if her/his colleagues help and support her/him (rarely, never), 0 otherwise (reference group)
Manager Support1	1 if her/his manager helps and supports her/him (always, most of the time), 0 otherwise
Manager Support2	1 if her/his manager helps and supports her/him (sometimes), 0 otherwise
Manager Support3	1 if her/his manager helps and supports her/him (rarely, never), 0 otherwise (reference group)
Colleagues Cooperation	If there is good cooperation between she/he and her/his colleagues (from 1 strongly agree to 6 strongly disagree)
Get on well Colleagues	If generally she/he gets on well with her/his work colleagues (from 1 strongly agree to 6 strongly disagree)
*Relationships outside the job*	
Volunteering	1 if she/he performs volunteer activities, 0 otherwise
Recreational activities	1 if she/he performs sporting, cultural or leisure activity outside her/his home, 0 otherwise
*Job sector*	
Private	1 if the interviewee works in the private sector, 0 otherwise
Public	1 if the interviewee works in the public sector, 0 otherwise
Other	1 if the interviewee works in a joint private-public organisation or company or the not-for-profit sector or an NGO or other, 0 otherwise (reference group)
*Kind of occupation*	
Armed forces	1 if the worker perform an armed forces occupation, 0 otherwise (reference group)
Managers	1 if the worker is a manager, 0 otherwise
Professionals	1 if the worker is a professional, 0 otherwise
Technicians	1 if the worker is a technician, 0 otherwise
Clerical	1 if the worker is a clerical support worker, 0 otherwise
Service sales	1 if the worker is a service and sales worker, 0 otherwise
Skilled agricultural forestry fish	1 if the worker is a skilled agricultural, forestry and fish worker, 0 otherwise
Craft trades	1 if the worker is craft and related trades worker, 0 otherwise
Plant machine	1 if the worker is a plant and machine operators, and assemblers, 0 otherwise
Elementary occupation	1 if the worker perform an elementary occupation, 0 otherwise

**Table 3:** Independent Variables: descriptive statistics

***Variable***	***Mean***	***Std. Dev.***	***Min***	***Max***
*Demographic*				
Male	.487	.499	0	1
Age1	.267	.442	0	1
Age2	.533	.498	0	1
Age3	.196	.397	0	1
Age4	.002	.043	0	1
Has a spouse or a partner	.611	.487	0	1
Has a child	.394	.488	0	1
Low level of education	.243	.429	0	1
Middle level of education	.678	.467	0	1
High level of education	.078	.269	0	1
End meet	3.13	1.273	1	6
*Job characteristics*				
Permanent job	.780	.414	0	1
Part time job	.265	.441	0	1
How many hours1	.161	.368	0	1
How many hours2	.691	.461	0	1
How many hours3	.116	.320	0	1
How many hours4	.030	.17	0	1
Health risk	.263	.440	0	1
Work affects health1	.120	.326	0	1
Work affects health2	.277	.447	0	1
Work affects health3	.601	.489	0	1
External Contacts	.256	.436	0	1
Work and Family	.829	.376	0	1
Trade Union	.532	.498	0	1
Job satisfaction	.855	.351	0	1
*Relationships on the job*				
Colleagues Support1	.752	.431	0	1
Colleagues Support2	.157	.364	0	1
Colleagues Support3	.089	.285	0	1
Manager Support1	.614	.486	0	1
Manager Support2	.206	.404	0	1
Manager Support3	.178	.383	0	1
Colleagues Cooperation	1.61	.778	1	5
Get on well Colleagues	1.529	.721	1	5
*Relationships outside the job*				
Volunteering	.285	.451	0	1
Recreational activities	.755	.429	0	1
*Job sector*				
Private	.700	.457	0	1
Public	.234	.423	0	1
Other	.064	.246	0	1
*Kind of occupation*				
Armed forces	.003	.062	0	1
Managers	.032	.177	0	1
Professionals	.123	.329	0	1
Technicians	.125	.330	0	1
Clerical	.121	.326	0	1
Service sales	.247	.431	0	1
Skilled agricultural forestry fish	.012	.112	0	1
Craft trades	.113	.316	0	1
Plant machine	.077	.268	0	1
Elementary occupation	.141	.348	0	1

### Methodology

The theoretical hypothesis concerning the association between *job stress* and interpersonal relationships on and outside of the job is tested using a standard ordered probit model that is largely used to examine discrete data of this type. In the ordered probit model, the probability of observing the outcome *j* corresponds to the probability that the estimated linear function, plus random error, is within the range of the cutpoints estimated for the outcome. The model is built around a latent regression of the following form:

1 $$ {y}_i\ast ={x}_i^{\hbox{'}}\beta +{\varepsilon}_i $$

where *x* and *β* are respectively the matrix of control variables and the vector of unknown parameters, *ε* is the error term, subscript *I* denotes an individual observation, and, as usual, y* is unobserved. We observe the following:

y = 0 if *y* ∗  ≤ 0.

y = 1 if 0 < *y* ∗  ≤ *μ*_1_.

y = J if *μ*_*J* − 1_ ≤ *y*∗.

indicating a form of censoring. Furthermore, is an unknown parameter to be estimated with *β*. We do not observe y* in the data. Rather, we observe the dependent variable, job stress.

In the ordered probit model, the interpretation of coefficients is very difficult and ambiguous [39], and neither the sign nor the magnitude of a coefficient provides information about the partial effects of a given explanatory variable. For this reason, we estimate marginal effects, which allow us to interpret the effect of the regressors on the dependent variable. Marginal effects serve as a measure of the expected direct change in the dependent variable as a function of the change in a certain explanatory variable while keeping all other covariates constant. The marginal effects of the regressors, which are expressed in terms of a change in the independent variables on the probability of “always” experiencing job stress and on the probability of “never” experiencing job stress, provide an indication of the magnitude of correlations between job stress and interpersonal relationships on and outside of the job.

## Results

Table 4 reports the marginal effects (*dx/dy*) of a change in the regressors on the probability of “always” (outcome 1) and “never” (outcome 5) experiencing job stress.

**Table 4:** The Marginal Effect (dx/dy) of a Change in the Regressors on the Probability of Experiencing Always and Never Stress

		Always			Never	
***Variable***	***dx/dy***	***SE***	***P > | z |***	***dx/dy***	***SE***	***P > | z |***
*Demographic*						
Male	−.026***	.003	0.000	.030***	.004	0.000
Age1	.021	.045	0.634	−.022	.043	0.602
Age2	.0199	.040	0.623	−.023	.048	0.630
Age3	.002	.041	0.944	−.003	.046	0.943
Has a spouse or a partner	.001	.003	0.636	−.002	.004	0.637
Has a child	−.003	.003	0.344	.004	.004	0.347
Middle level of education	.008*	.004	0.052	−.010*	.005	0.060
High level of education	.022***	.008	0.009	−.022***	.007	0.002
End meet	.003 **	.001	0.041	−.003**	.001	0.042
*Job characteristics*						
Permanent job	.010**	.004	0.026	−.0124**	.005	0.037
Part time job	−.006	.004	0.143	.007	.005	0.157
How many hours2	.012**	.005	0.023	−.015**	.007	0.031
How many hours3	.045***	.009	0.000	−.040***	.006	0.000
How many hours4	.073***	.019	0.000	−.052***	.008	0.000
Health risk	.041***	.005	0.000	−.040***	.004	0.000
Work affects health1	.009*	.005	0.080	−.009*	.005	0.063
Work affects health2	.085***	.006	0.000	−.074***	.004	0.000
External Contacts	.020***	.004	0.000	−.021***	.003	0.000
Work and Family	−.050***	.005	0.000	.044***	.004	0.000
Trade Union	.013***	.003	0.000	−.016***	.004	0.000
Job satisfaction	−.054***	.007	0.000	.046***	.004	0.000
*Relationships on the job*						
Colleagues Support1	.025***	.005	0.000	−.033***	.008	0.000
Colleagues Support2	.027***	.008	0.001	−.027***	.007	0.000
Manager Support1	−.014***	.005	0.006	.0159***	.005	0.005
Manager Support2	.000	.005	0.986	−.000	.005	0.986
Colleagues Cooperation	.009***	.002	0.000	−.011***	.003	0.000
Get on well Colleagues	.014***	.002	0.000	−.016***	.003	0.000
*Relationships outside the job*						
Volunteering	−.001	.003	0.659	.001	.003	0.661
Recreational activities	−.009**	.004	0.039	.010**	.004	0.030
*Job sector*						
Private	.020***	.005	0.001	−.024***	.007	0.001
Public	.005	.006	0.405	−.006	.007	0.393
*Kind of occupation*						
Managers	.048	.036	0.184	−.039*	.021	0.062
Professionals	.012	.027	0.655	−.013	.027	0.629
Technicians	.001	.025	0.963	−.001	.029	0.963
Clerical	−.004	.024	0.864	.005	.030	0.868
Service sales	−.013	.023	0.576	.016	.031	0.601
Skilled agricultural forestry fish	−.049***	.014	0.001	.104*	.056	0.064
Craft trades	−.039**	.018	0.028	.063	.039	0.109
Plant machine	−.035*	.018	0.054	.055	.039	0.158
Elementary occupation	−.045***	.016	0.006	.077*	.041	0.063
Austria	.038***	.012	0.003	−.034***	.008	0.000
Belgium	.000	.008	0.936	−.000	.009	0.936
Denmark	−.048***	.005	0.000	.092***	.016	0.000
Finland	−.038***	.006	0.000	.063***	.014	0.000
France	−.033***	.007	0.000	.051***	.015	0.001
Germany	.006	.008	0.442	−.007	.009	0.422
Greece	.010	.011	0.342	−.011	.011	0.301
Ireland	−.016*	.009	0.072	.021	.013	0.118
Italy	−.032***	.007	0.000	.051***	.015	0.001
Luxemburg	.010	.013	0.435	−.011	.013	0.393
Netherlands	−.044***	.006	0.000	.082***	.020	0.000
Portugal	−.008	.010	0.372	.011	.013	0.408
Spain	−.020***	.007	0.008	.026**	.011	0.021
Switzerland	−.010	.008	0.248	.012	.012	0.288

***stat. Signf. at 1%, ** stat. Signf. at 5%, and * stat. Signf. at 10%

Marginal effects are often used in several disciplines (such as economics) since they are likely to provide a good assessment to the amount of change in Y that will be produced by a 1-unit change in X_k_. Therefore, marginal effects provide information on how modification in a reply is related to modification in a covariate. The marginal effects for categorical variables indicate how P(Y = 1) is predicted to change as X_k_ varies from 0 to 1 keeping all other X_s_ equal. This can be quite helpful, explanatory, and simple to recognize [39].

It is essential to clarify that the findings of the econometric analyses, as part of a cross-sectional study (no panel dimension is available), define correlations rather than cause-and-effect relations between job stress and relationships on and outside of the job, and association does not indicate causation. We do not find a clear causal relationship in one direction or the other: causation may go in both directions, with the workers who do not report job stress having more opportunities to interact with others on and outside of the job and with interpersonal relationships on and outside of the job influencing workers’ levels of job stress.

According to the literature [9, 40], estimated marginal effects show that relationships on and outside of the job influence job stress. In particular, the results for the variables of interest show that:

- Reporting being helped and supported by colleagues always and most of the time is associated with a 2.5% higher probability of always experiencing stress and with a 3.3% lower probability of never experiencing stress than not receiving help and support from colleagues. Reporting being helped and supported by colleagues sometimes is associated with a 2.7% higher probability of always experiencing stress and with a 2.7% lower probability of never experiencing stress than not receiving help and support from colleagues.
- Reporting being helped and supported by one’s manager always or most of the time is associated with a 1.4% lower probability of always experiencing stress and with a 1.6% higher probability of never experiencing stress than not receiving help and support from one’s manager.
- As cooperation with colleagues worsens, the probability of always reporting job stress is expected to increase by 0.9%, while the probability of never reporting stress is expected to decrease by 1.1%.
- As relationships with colleagues worsen, the probability of always being stressed is expected to increase by 1.4%, and the probability of never reporting stress is expected to decrease by 1.6%.
- Performing tasks that involve external contact (visiting customers, patients or clients or working from home) is associated with a 2% higher probability of always being stressed and with a 2.1% lower probability of never being stressed than not engaging in external work activities.
- Practising recreational (sport, cultural and leisure) activities is associated with a 0.9% lower probability of always reporting job stress and with a 1% higher probability of never reporting job stress than not being involved in such activities.
- The possibility of scheduling working hours in relation to family and external commitments is associated with a 5% lower probability of always experiencing stress and with a 4.4% higher probability of never reporting job stress than being unable to balance family life with work responsibilities.

## Discussion

As expected, our results show some significant correlations between job stress and maintaining interpersonal contacts on and outside of the job (that does not mean causation, i.e. causation may apply to both directions): in some cases, interacting with others may be important in counteracting negative effects of unfavourable working conditions on the occurrence of job stress. This is the case for interactions with one’s manager: help and support provided by one’s manager decreases the probability of being stressed at work [17, 34]. According to the literature (see, among others [33, 34, 41]), receiving help and support from co-workers is likely to increase the probability of job stress occurrence. The reason for this probably lies in the different nature of the two relationships: while interactions with co-workers can become a cause of frustration (possibly because competition and conflicts may arise among peers), interactions with one’s manager are not (likely because managers are have a different definite role, which, for instance, could involve job promotion). However, maintaining cooperation and getting on well with colleagues decrease the probability of experiencing stress, confirming the positive and gratifying features of contact with co-workers reported by the literature (see among others [26, 42]). These contrasting results are rooted in the different connotations of social support originating from cooperation and congeniality. While help and support can degenerate in periods of conflict due to competition, cooperation and congeniality, which are likely spontaneous, may make workers feel less alone, as they know that they can count on their colleagues for general advice and discussion and for assistance with how to do their jobs. According to the literature [34], relationships forged through performing activities involving external contact with clients and patients seem to be less conducive to managing stress and possibly because such jobs often involve performing challenging tasks for persons with various problems (e.g., interactions with disable people).

As expected, relationships forged through recreational activities, which are generally performed within groups, are likely to counteract unpleasant effects of negative working conditions and to decrease the probability of always reporting job stress: workers who practise sports and who participate in cultural and leisure events have a higher probability of not experiencing stress than workers who do not. This difference likely occurs because places where those recreational activities are practised become networks in which it is possible to share emotions and problems. Speaking with others and knowing that others will provide support in times of need are likely to mitigate job stress.

The possibility of handling family relationships could serve as a good resource in reducing job stress, and since striking a proper balance between work and family is not easy to achieve, when it becomes difficult to balance work with family commitments, this mismatch can become a source of stress [9, 40]. Our results show that workers who balance work with family responsibilities experience less job stress than workers who do not.

Regarding the demographic variables, according to the literature [17], males are less likely (2.6%) than females to report always experiencing job stress: women face workplace sexism more than men, have more familial responsibilities, are required to prove that they are as good as men at their jobs; women also often receive less pay. In line with the literature (see among other [43]), those with moderate and high levels of education are associated with a higher probability of always reporting job stress than those with low levels of education. As expected, when a worker’s total household monthly income is insufficient to cover personal costs, the probability of always experiencing stress increases. In terms of job characteristics, workers with permanent jobs have a higher (1%) probability of reporting job stress than workers with fixed-term contracts: these results are in line with the literature [44] and can be attributed to the fact that permanent workers have more responsibilities than temporary workers. As expected, increasing working hours are associated with a higher probability of experiencing job stress [17]. Believing that one’s health or safety is at risk due to one’s work increases the probability of reporting always experiencing stress (4%). As work affects one’s health both positively and negatively, it can increase the probability (respectively 0.9 and 8.5%) of reporting job stress. Contrary to the literature (see, for instance [45]) stating that “trade unions are essential in the prevention of stress at work … [40]”, our results show that trade union presence within an organization increases the probability of reporting job stress. Workers who are satisfied with their working conditions have a 5.4% lower probability of always reporting stress, and this is likely the case because experiencing job satisfaction can improve one’s level of self-confidence and communication in the workplace; in turn, levels of psychological distress are decreased. Those who work in the private sector have a higher probability of always reporting stress than those working in other sectors (nonprofit, NGO, and joint private public organizations). This is likely the case because organizations of the two sectors are presumed to function differently, with the latter being more likely than private enterprises to provide work environments in which the quality of work and interpersonal relationships is valued. Regarding the type of occupation, our results show that skilled workers and workers of the agricultural, forestry, fishing, trade plant machine and elementary occupations have a lower probability of reporting stress than armed forces workers. Country dummies show that workers in Denmark, Finland, France, Ireland, Italy, the Netherlands, and Spain have a lower probability of always reporting job stress than workers in the UK, while Austrian workers show a higher probability always reporting job stress.

## Conclusions

Job stress is a by-product that has emerged from rapid changes in working conditions registered over the last decades in globalized economies. Coping with job stress should be a priority in the agendas of national economies since stress negatively affects work performance with consequences at the individual and organizational levels. Work-related stress is associated with risky behavioural outcomes that can include high alcohol consumption [46] and difficulties managing strong relationships with one’s partners and children [47]. In addition, job stress is one of the main causes of several diseases, including coronary heart disease [13]. All of these trends stand in contrast with overall aims in the workplace to help individuals and organizations flourish [48].

The main aim of this paper was to examine the link between job stress and interpersonal relationships on and outside of the job based on data taken from the last European Working Condition Survey (a cross-sectional survey). The paper has some limitations. The theoretical framework within which the paper places is the “demand-control-support” model, however, our response variable, “job stress”, has been chosen given the availability of information in the EWCS6 (2017) and therefore, it is likely to be inconsistent to the model. Moreover, job stress is a self-rated/subjective indicator about which questions of reliability have been raised (see the Dependent variable section). In addition, we were not able to establish the direction of causality between job stress and interpersonal relationships, however, the present work contributes new evidence to the literature on job stress. Our results show that interpersonal relationships on and outside of the job can be considered valuable resources that, when available to an individual, are useful for managing stress created by workplace stressors (high demands and low control): “extensive social support is essential in preventing stress at work” [40].

A strength of the paper lies in the sample analysed, which includes a large set of countries such that the results provide a broad account of the relationship between job stress and relationships on and outside of the job. Further, the aggregation of countries (EU15) characterized by different work-related features could be a limitation of this study since different countries are characterized by dissimilar working environments. However, countries dummies were included in the empirical analysis.

As observed, job stress is a very multifaceted and generalized phenomenon, affecting not only the health and wellbeing of people at work but also economies and society at large. However, job stress is still considered a personal challenge rather than an organizational issue. Thus, more research is needed to identify workplace stressors and to promote healthy work environments.

## Data Availability

The datasets used and/or analyzed during the current study are from the European Foundation for the Improvement of Living and Working Conditions. (2017). *European Working Conditions Survey, 2015*. [data collection]. *4th Edition.* UK Data Service. SN: 8098, 10.5255/UKDA-SN-8098-4 The data were accessed and downloaded via the UK Data Service. The terms of service for the website from which data was collected were complied.
